# Molecular mechanisms for DNA methylation defects induced by ICF syndrome‐linked mutations in DNMT3B

**DOI:** 10.1002/pro.5131

**Published:** 2024-09-18

**Authors:** Chao‐Cheng Cho, Cheng‐Yin Fei, Bo‐Chen Jiang, Wei‐Zen Yang, Hanna S. Yuan

**Affiliations:** ^1^ Institute of Molecular Biology, Academia Sinica Taipei Taiwan, ROC; ^2^ Graduate Institute of Biochemistry and Molecular Biology National Taiwan University Taipei Taiwan, ROC

**Keywords:** crystal structure, DNA methylation, epigenetic modification, human disease

## Abstract

DNA methyltransferase 3B (DNMT3B) plays a crucial role in DNA methylation during mammalian development. Mutations in DNMT3B are associated with human genetic diseases, particularly immunodeficiency, centromere instability, facial anomalies (ICF) syndrome. Although ICF syndrome‐related missense mutations in the DNMT3B have been identified, their precise impact on protein structure and function remains inadequately explored. Here, we delve into the impact of four ICF syndrome‐linked mutations situated in the DNMT3B dimeric interface (H814R, D817G, V818M, and R823G), revealing that each of these mutations compromises DNA‐binding and methyltransferase activities to varying degrees. We further show that H814R, D817G, and V818M mutations severely disrupt the proper assembly of DNMT3B homodimer, whereas R823G does not. We also determined the first crystal structure of the methyltransferase domain of DNMT3B‐DNMT3L tetrameric complex hosting the R823G mutation showing that the R823G mutant displays diminished hydrogen bonding interactions around T775, K777, G823, and Q827 in the protein‐DNA interface, resulting in reduced DNA‐binding affinity and a shift in sequence preference of +1 to +3 flanking positions. Altogether, our study uncovers a wide array of fundamental defects triggered by DNMT3B mutations, including the disassembly of DNMT3B dimers, reduced DNA‐binding capacity, and alterations in flanking sequence preferences, leading to aberrant DNA hypomethylation and ICF syndrome.

## 1 INTRODUCTION

ICF syndrome, which stands for Immunodeficiency, Centromere instability, Facial anomalies syndrome, is a rare autosomal recessive disease that was first discovered about four decades ago (Hulten, 1978; Maraschio et al., 1988; Tiepolo et al., 1979). The syndrome is characterized by a range of clinical features, including weakened immune systems with low levels of immunoglobulins, developmental delay, centromere instability, and facial anomalies (Jeanpierre et al., 1993; Smeets et al., 1994). ICF syndrome has been linked to defects in four genes, namely *DNMT3B*, *ZBTB24*, *CDCA7*, and *HELLS* (Boogaard et al., 2017; Ehrlich et al., 2006; Vukic & Daxinger, 2019). The primary cause of ICF is homozygous or compound heterozygous mutations in the *DNMT3B* gene that encodes DNA methyltransferase 3B (DNMT3B), which catalyzes cytosine methylation during embryonic development (Gagliardi et al., 2018; Okano et al., 1999; Xu et al., 1999). Approximately 60% of ICF syndrome patients carry a mutation in *DNMT3B*, classified as Type 1 ICF (ICF1), leading to DNA hypomethylation in classical satellites 2 and 3 at centromeric regions of chromosomes 1, 9, and 16, resulting in centromeric instability (Kiaee et al., 2021). Genome‐wide DNA methylation and transcriptomic studies of ICF patients have revealed an approximate 40% reduction in global methylation levels, with the most profound methylation changes occurring in inactive heterochromatic regions, satellite repeats, intragenic regions of highly transcribed genes, and transposons (Gatto et al., 2017; Heyn et al., 2012). These findings strongly indicate that DNMT3B dysfunction and aberrant DNA methylation play a pivotal role in ICF syndrome development.

**FIGURE 1:**
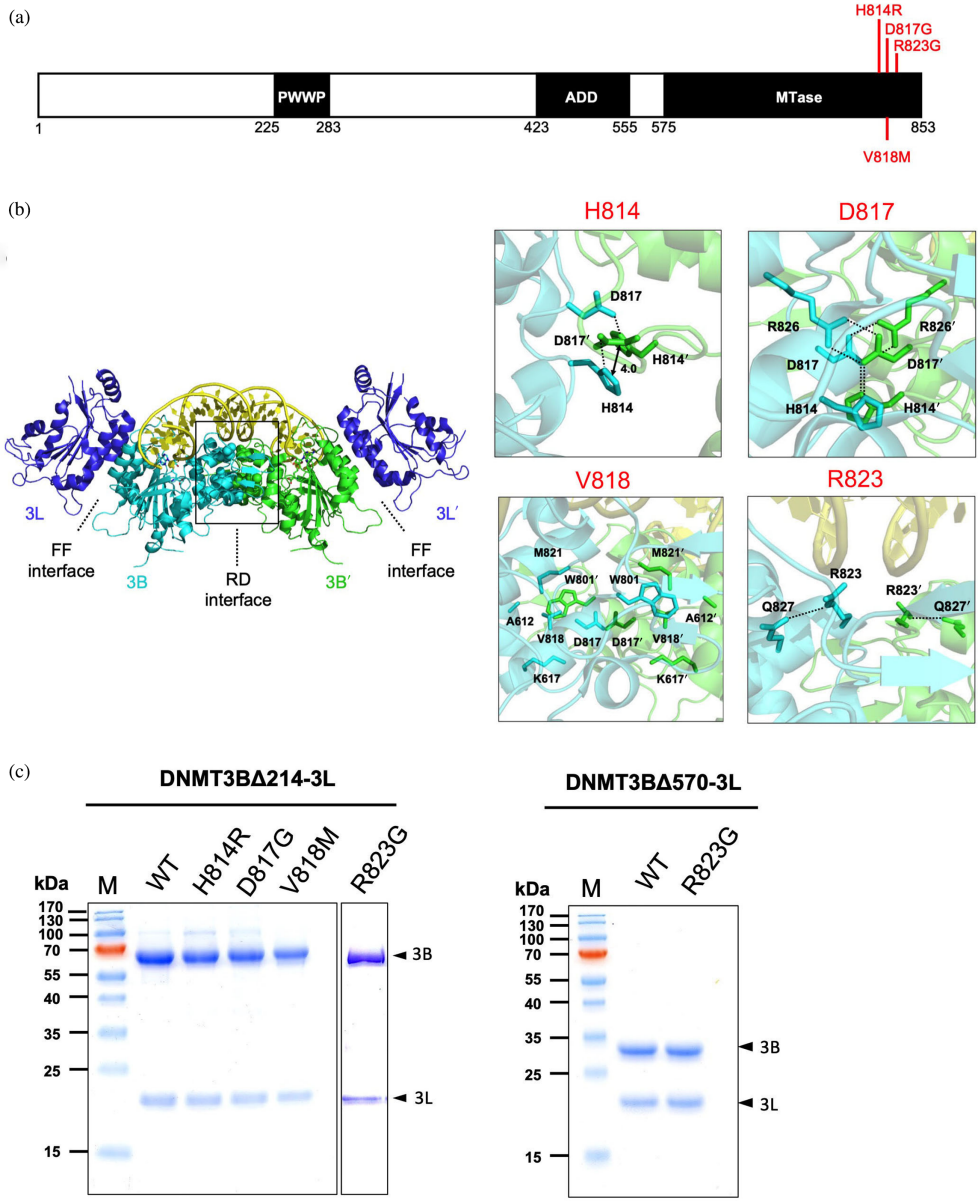
Four immunodeficiency, centromere instability, facial anomalies (ICF) syndrome‐associated DNA methyltransferase 3B (DNMT3B) mutations are located within the DNMT3B homodimeric interface of the tetrameric DNMT3B‐3L complex. (a) Schematic diagram showing the domain arrangement of DNMT3B. The ICF syndrome‐linked mutations located in the DNMT3B homodimeric interface are marked in red. (b) Locations of the four ICF‐linked mutations in the DNMT3B homodimeric interface (RD interface) in the crystal structure of the DNMT3B‐3L‐DNA complex (PDB code: 6KDA) (Lin et al., 2020). Enlarged views of each mutational residue are shown as stick models in the panels at right. Residues in the DNMT3B′ protomer are followed by the ′ notation. (c) Wild‐type (WT) DNMT3BΔ214‐3L and the corresponding mutant complexes (H148R, D817G, V818M, and R823G), as well as the WT DNMT3BΔ570‐3L and corresponding R823G mutant complexes, were purified to high homogeneity, as revealed by SDS–PAGE of each recombinant protein. M indicates the lane of molecular‐weight markers. 3B and 3L represent DNMT3B and DNMT3L, respectively (indicated by arrowheads).

DNMT3B and DNMT3A both act as de novo DNA methyltransferases playing key roles during early mammalian development and maturation of germ cells (Jia et al., 2007; Lin et al., 2020; Lyko, 2018; Suetake et al., 2004). DNMT3B and DNMT3A share a similar domain organization, featuring an N‐terminal regulatory proline‐tryptophan‐tryptophan‐proline (PWWP) domain and an ATRX‐DNMT3‐DNMT3L (ADD) domain, followed by a C‐terminal catalytic methyltransferase (MTase) domain (Figure 1a). The majority of ICF‐related missense mutations in DNMT3B occur within the MTase domain, including A603T (Okano et al., 1999); V726G (Hansen et al., 1999); S282P, and R832Q (Shirohzu et al., 2002); A585V, V606A, V699G, A766P, H814R, and V818M (Wijmenga et al., 2000); V606A, A766P, H814R, R823G, and R840Q (Gao et al., 2020; Xie et al., 2006). Crystal structures of the MTase domain of DNMT3B in complex with the inactive MTase domain of the stimulatory protein DNMT3L have been reported previously (Gao et al., 2020; Lin et al., 2020). The structure of this hetero‐tetrameric DNMT3B‐3L complex reveals a homodimeric DNMT3B assembly (with each monomer interacting via what is termed the “RD interface”) in a linear arrangement of DNMT3L‐DNMT3B‐DNMT3B‐DNMT3L, with the inactive MTase domain of DNMT3L interacting with DNMT3B via the so‐called “FF interface” (see Figure 1b). Notably, two loops of the DNMT3B homodimer, the catalytic and target recognition domain (TRD) loops, are responsible for clamping CpG sites of the DNA minor and major grooves, respectively. The TRD loop recognizes the guanine base and interacts with the base located at +1 flanking position, whereas the catalytic loop flips out the cytosine base for methylation. The crystal structure of the DNMT3B‐3L‐DNA complex has served as a foundation for elucidating the potential enzymatic or complex assembly defects of most ICF syndrome‐linked mutations, such as those affecting cofactor *S*‐adenosylmethionine binding (A585V, A603T, and V606A), stabilization of protein folding (A603T, V726G, A766P, R840Q), and DNA binding (R823G). Moreover, mutations located in the FF interface between DNMT3B and DNMT3L, including R670Q and L664P/T, have been shown to impair both DNMT3B homo‐oligomeric and DNMT3B‐3L hetero‐tetrameric assemblies, MTase activity, and heterochromatin targeting of DNMT3B, ultimately resulting in reduced global DNA methylation in cells (Gao et al., 2022).

The mutations situated within the FF interface of the DNMT3B‐3L complex have been extensively investigated (Gao et al., 2022). However, the ICF syndrome‐associated mutations—including H814R, D817G, V818M, and R823G—which are positioned near or directly within the RD interface, have not been thoroughly explored. In the crystal structure of DNMT3B‐3L‐DNA complex, the H814 side‐chain stacks with that of the H814′ of the neighboring protomer and makes a hydrogen bond with D817′ (where ′ represents a residue in the other protomer of the homodimer); D817 forms salt bridges with R826′ and a hydrogen bond with H814′; V818 is involved in hydrophobic interactions with W801′, and R823 forms a hydrogen bond with Q827 (Figure 1b). Thus, all of these residues may contribute to dimer assembly and stabilization. The ICF‐associated R823G mutation in DNMT3B is aligned at the same position with the most prevalent acute myeloid leukemia (AML)‐associated mutation R882H in DNMT3A, indicating the importance of this arginine residue in maintaining the MTase activity for both DNMT3A and DNMT3B (Holz‐Schietinger et al., 2012; Ley et al., 2010). Indeed, mutation of R882H in DNMT3A compromises enzymatic activity, CpG specificity, cooperativity, and flanking sequence preference (Emperle et al., 2019; Gao et al., 2022; Mack et al., 2022; Norvil et al., 2020). Particularly, when compared with wild‐type (WT) DNMT3A, R882H mutant has significantly altered flanking sequence preferences, which directly affect cellular methylation patterns and are correlated with misregulations of several genes with putative connections to AML (Emperle et al., 2019; Mack et al., 2022). In the crystal structures of DNMT3B‐3L‐DNA complex, R823 is located at the protein‐DNA interface, positioned proximal to the phosphate group of the +4 flank site (Gao et al., 2020; Lin et al., 2020). The R823G mutation in DNMT3B led to defects in DNA dissociation (Moarefi & Chedin, 2011), whereas the R823A mutation altered the enzyme's flanking sequence preference, enhancing its preference for methylation of GGG sequences at flank positions +1 to +3 (Dukatz et al., 2020). The H814R mutation of DNMT3B has been shown to disrupt dimerization. Additionally, the D817G mutation of DNMT3B was found to reduce its methylation activity, with mice carrying this mutation exhibiting phenotypes reminiscent of those displayed by ICF patients, including hypomethylation of repetitive sequences, low body weight, distinct craniofacial anomalies, and apoptotic T cell death (Ueda et al., 2006).

In this study, we systematically assessed the impact of these ICF syndrome‐associated mutations in the RD interface on the assembly, as well as enzymatic and DNA‐binding activities, of the DNMT3B‐3L complex, together with their impacts on structural folding and methylation sequence preferences. Our results demonstrate that all of the RD interface mutations we assessed compromise both the enzymatic and DNA‐binding activities of the DNMT3B‐3L complex. Notably, the H814R, D817G, and V818M mutations severely disrupt oligomerization of the DNMT3B‐3L hetero‐tetramer, whereas the R823G mutation does not significantly affect this process. To gain deeper insights into the R823G mutation, we determined the first crystal structure of an ICF syndrome‐linked mutant of DNMT3B, that is the MTase domain of the DNMT3B‐3L hetero‐tetramer containing the R823G mutation at a resolution of 3.0 Å, demonstrating the underlying mechanism for its defects in DNA binding and methylation. Our structural analysis reveals that substitution of R823 with glycine impairs the hydrogen bonding between R823 and Q827, as well as between Q827 and the residues (T775 and K777) located in the TRD loop involved in DNA binding, leading to reduced DNA‐binding affinity. We also uncover that the R823G mutation exerts a notable influence on DNMT3B's flanking sequence preference, displaying an increased preference for guanine at the +1 to +3 flanking positions. Thus, our findings reveal the multiple molecular consequences of these DNMT3B mutations in terms of their impacts on epigenetic complex assembly, DNA binding, methylation activity, and sequence preference. These insights provide a comprehensive understanding on how these mutations ultimately contribute to the observed hypomethylation in individuals with ICF syndrome.

## 2 RESULTS

### 2.1 H814R, D817G, and V818M mutations disrupt DNMT3B dimerization

To investigate the impacts of ICF syndrome‐associated mutations in the RD interface of the DNMT3B homodimer, we introduced H814R, D817G, V818M, and R823G mutations into a construct expressing N‐terminal tail‐truncated DNMT3B (DNMT3BΔ214, residues 215–853) that still hosts the PWWP, ADD, and MTase domains (Figure 1a). The resulting constructs expressed stable recombinant proteins of WT or mutated DNMT3BΔ214, which was co‐purified with the DNA MTase‐like domain of DNMT3L (residues 179–379) as a DNMT3BΔ214‐3L complex to high homogeneity as shown by the SDS–PAGE (Figure 1c). The R823G mutation was also introduced into a shorter construct solely hosting the MTase domain (DNMT3BΔ570, residues 571–853), which was also co‐purified with the DNA MTase‐like domain of DNMT3L (residues 179–379) as a DNMT3BΔ570‐3L complex. SDS–PAGE confirmed the high homogeneity of the WT and mutated DNMT3BΔ570‐3L complexes (Figure 1c).

**FIGURE 2:**
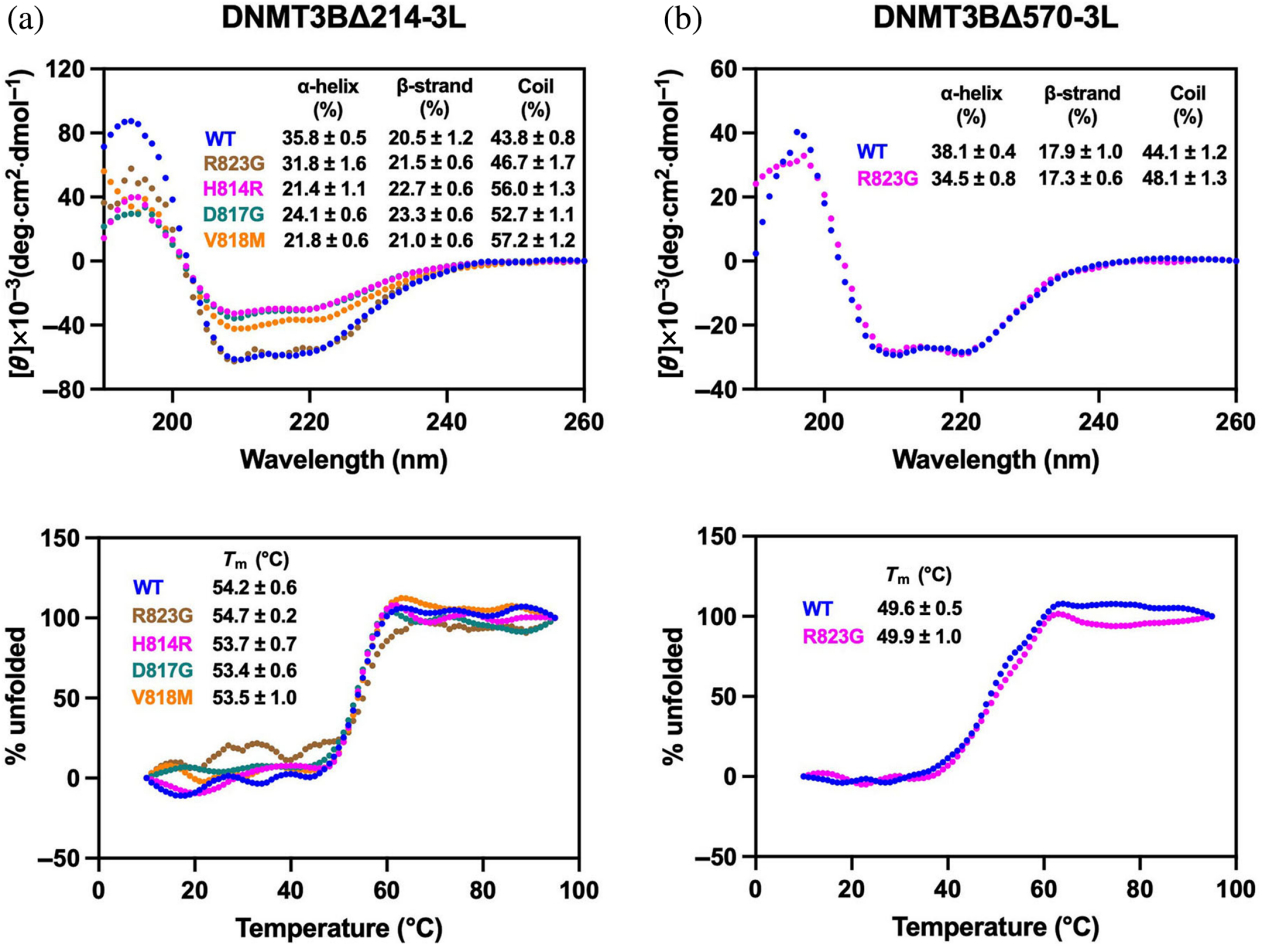
Immunodeficiency, centromere instability, facial anomalies (ICF) syndrome‐associated mutations H148R, D817G, and V818M in DNA methyltransferase 3B (DNMT3B) disturb the overall folding of the DNMT3B‐3L complexes. (a) Circular dichroism (CD) spectra of wild‐type (WT) DNMT3BΔ214‐3L and the mutant complexes (400 nM) recorded from 260 to 190 nm in the buffer containing 20 mM phosphate at pH 7.4. The inset shows the predicted percentages of α‐helices, β‐sheets, and random coils. Lower panel displays the thermal denaturation profile of DNMT3BΔ214‐3L complexes by monitoring the CD signal at 208 nm upon gradually increasing the temperature from 10 to 95°C. The inset shows the melting temperature (*T*_m_) derived from the first derivative of the data. (b) CD spectra of WT DNMT3BΔ570‐3L and the R823G mutant complex (400 nM) recorded from 260 to 190 nm in the buffer containing 20 mM phosphate at pH 7.4. The inset shows the predicted percentages of α‐helices, β‐sheets, and random coils. Lower panel displays the thermal denaturation profile of WT DNMT3BΔ570‐3L and the R823G mutant complex by monitoring the CD signal at 208 nm upon gradually increasing the temperature from 10 to 95°C. The inset shows the *T*_m_ derived from the first derivative of the data. DNMT3B, DNA methyltransferase 3B.

We first assessed overall folding of these DNMT3B‐3L complexes by means of circular dichroism (CD). The CD profile of the DNMT3BΔ214‐3L complex hosting the R823G mutation was similar to that of WT complex, and the estimated secondary structure compositions of the two complexes were close (within a 4% difference) to each other (Figure 2a). Similarly, we observed no significant difference in the CD profiles or secondary structure compositions of WT DNMT3BΔ570‐3L and the R823G mutant, supporting that the R823G mutation does not exert a major impact on structural folding of the DNMT3BΔ570‐3L complex (Figure 2b, upper panel).

However, the CD profiles of the DNMT3BΔ214‐3L complexes hosting the H814R, D817G, or V818M mutation measured at the same concentration as that of the WT complex presented a reduction in signal intensity relative to the WT complex, indicating a reduced amount of secondary structure elements in these mutant complexes (Figure 2a, upper panel) (Nomura et al., 2013). Indeed, the secondary structure analysis revealed a ca. 12%–14% reduction in α‐helices, and a ca. 9%–13% increase in random coils in the H814R, D817G and V818M mutant complexes compared to WT DNMT3BΔ214‐3L (Figure 2a). Moreover, we observed that the mutant proteins tended to precipitate over time. Therefore, the reduced CD signal may also be attributed to the lower concentrations of mutant proteins in solutions. The thermal melting temperature of WT and any of the mutant complexes were similar (Figure 2a,b, lower panels), indicating that these mutations did not alter the thermal stability of the well‐folded DNMT3B‐3L complexes. Overall, our CD analysis indicates that the H814R, D817G, and V818M mutations reduce secondary structures of the DNMT3BΔ214‐3L complex, whereas the R823G mutation exerts only a subtle impact on the overall structure of DNMT3BΔ214‐3L and DNMT3BΔ570‐3L complexes.

**FIGURE 3:**
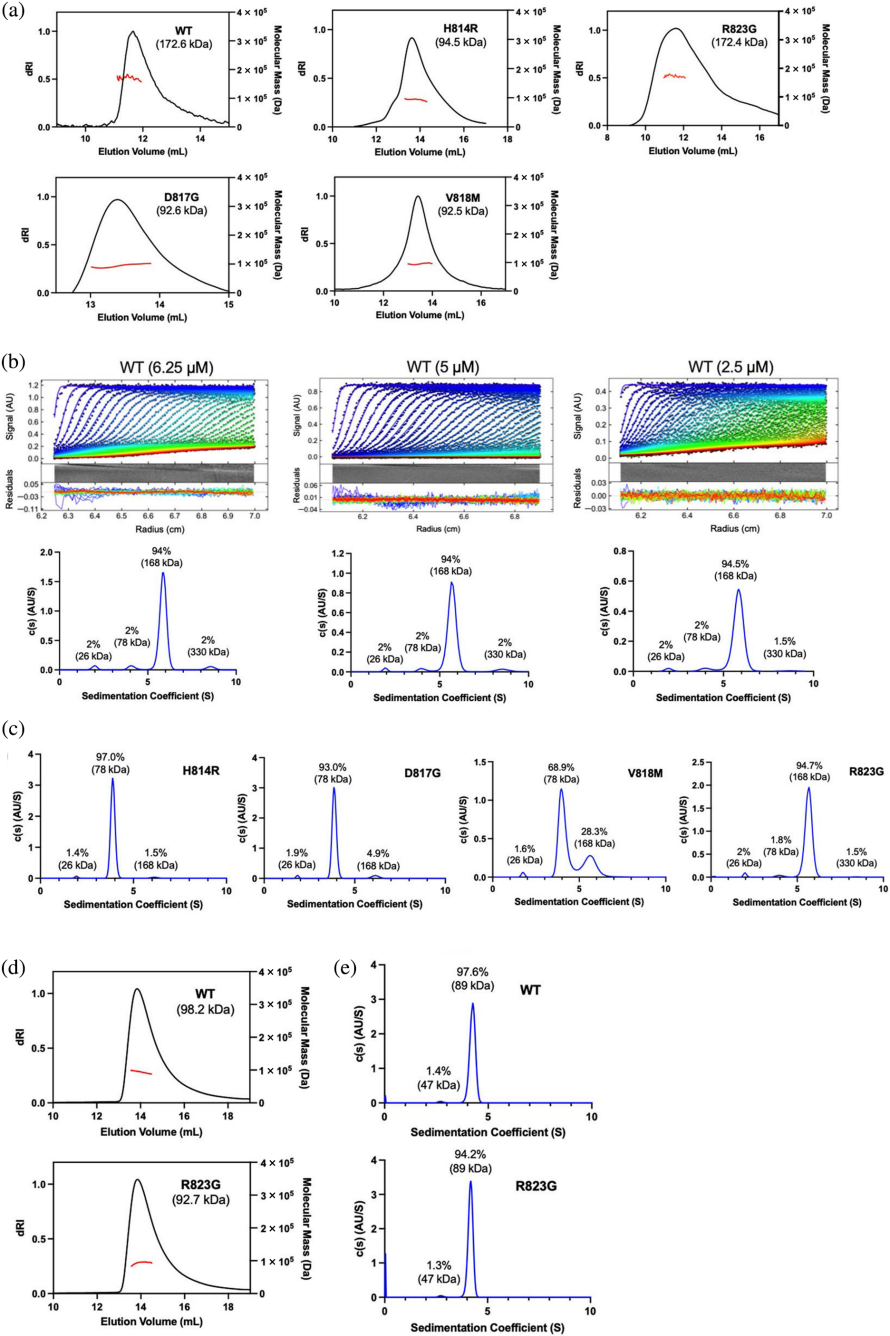
Mutations of H148R, D817G, and V818M at the DNA methyltransferase 3B (DNMT3B) dimeric interface disrupt DNMT3B‐3L tetramer assembly. (a) The molecular weights of wild‐type (WT) and mutated DNMT3BΔ214‐3L complexes were determined by size‐exclusion chromatography coupled to multi‐angle light scattering (SEC‐MALS). The SEC elution profiles are displayed in black lines, whereas the estimated MW from MALS are shown in red lines. The theoretical MW of the tetrameric DNMT3BΔ214‐3L complex is 192.2 kDa. (b) The sedimentation velocity experiments (upper panels) were performed by analytical ultracentrifugation (AUC) for the WT DNMT3BΔ214‐3L complex at different concentrations (6.25, 5.0, and 2.5 μM). Sedimentation coefficient distribution profiles (bottom panels) reveal the proportions and molecular weights of the DNMT3BΔ214‐3L complex indicated above each peak. (c) Sedimentation coefficient distribution profiles for the four mutants hosting in the DNMT3BΔ214‐3L complex. (d) The molecular weights of WT DNMT3BΔ570‐3L and R823G mutant complexes were determined by SEC‐MALS. (e) Sedimentation coefficient distribution profiles of WT and mutant DNMT3BΔ570‐3L complexes from AUC analyses.

Next, we examined oligomerization of the DNMT3B‐3L complex by means of size‐exclusion chromatography coupled to multi‐angle light scattering (SEC‐MALS). The SEC‐MALS‐determined molecular weights (red lines in Figure 3a) of the WT DNMT3BΔ214‐3L complex and the one hosting the R823G mutation measured at the same concentrations of 25 μM were 172.6 and 172.4 kDa, respectively, supporting a hetero‐tetrameric assembly (compared to a theoretical MW of the tetrameric complex of 192.2 kDa). Notably, the estimated MW for the complexes hosting the H814R, D817G, or V818M mutations measured at the concentrations of ca. 25 μM were significantly reduced to 94.5, 92.6, and 92.5 kDa, respectively (Figure 3a). Thus, the H814R, D817G, and V818M mutations likely prompt the DNMT3BΔ214‐3L tetrameric complex to dissociate into heterodimers (theoretical MW of the dimeric complex: 96.1 kDa).

To gain further insights, we examined the size of the complexes using analytical ultracentrifugation (AUC). For WT DNMT3BΔ214‐3L complex centrifuged at different concentrations ranging from 6.25 to 2.5 μM, the AUC profiles revealed a major peak with an estimated MW of 168 kDa (94%) and a minor peak with an estimated MW of 78 kDa (2%), reflecting an abundant hetero‐tetrameric assembly and a smaller heterodimeric population (Figure 3b). The consistent AUC profiles observed across various concentrations (ranging from 6.25 to 2.5 μM) of the DNMT3BΔ214‐3L complex imply the formation of stable populations of tetramers and dimers within this concentration range. Two minor peaks were also observed at 330 kDa (1.5%–2%), and 26 kDa (2%), indicative of respective aggregation (of two tetramers) and degradation of the protein samples in AUC experiments during overnight centrifugation.

The AUC profile for the DNMT3BΔ214‐3L complex hosting the R823G mutation revealed similar populations to the WT complex: a major peak with a MW of 168 kDa (94.7%) and a minor peak with a MW of 78 kDa (1.8%), indicating a major tetrameric and a minor dimeric complex (Figure 3c). In contrast, for the three mutant complexes in a concentration of 5 μM, AUC revealed a major peak of 78 kDa for the H814R (97.0%), D817G (93.0%), and V818M (68.9%) indicating that these three mutations disrupted DNMT3B dimer formation and generated primarily a smaller heterodimeric complex (Figure 3c). Notably, we observed a minor peak with an identical MW of 168 kDa to the WT tetrameric complex—representing 1.5%, 4.9%, and 28.3% of the H814R, D817G, and V818M mutant complex populations, respectively—so these mutations in the RD interface did not completely abrogate hetero‐tetrameric complex assembly.

For the R823G mutation hosting in the DNMT3BΔ570‐3L complex, SEC‐MALS revealed a slightly lower MW of 92.7 kDa for the R823G mutant compared to an estimated MW of 98.2 kDa (theoretical MW: 112.5 kDa) for the WT complex (Figure 3d). AUC further revealed that 97.6% of WT DNMT3BΔ570‐3L complex and 94.2% of the R823G mutant complex were assembled into hetero‐tetramers (Figure 3e). Thus, unlike the H814R, D817G, and V818M mutations, R823G substitution only slightly disrupts tetrameric assembly of the epigenetic DNMT3B‐3L complex. In summary, our findings from the SEC‐MALS and AUC experiments underscore the disruptive effects of the H814R, D817G, and V818M mutations on formation of the DNMT3BΔ214‐3L heterotetramer. Consequently, these DNMT3BΔ214‐3L mutants primarily formed heterodimers, with a residual population of heterotetramers. In contrast, the R823G mutation does not significantly alter heterotetrameric assembly of the DNMT3BΔ214‐3L and DNMT3BΔ570‐3L complexes.

### 2.2 ICF‐linked mutations in DNMT3B impair DNA‐binding and methylation activities

**FIGURE 4:**
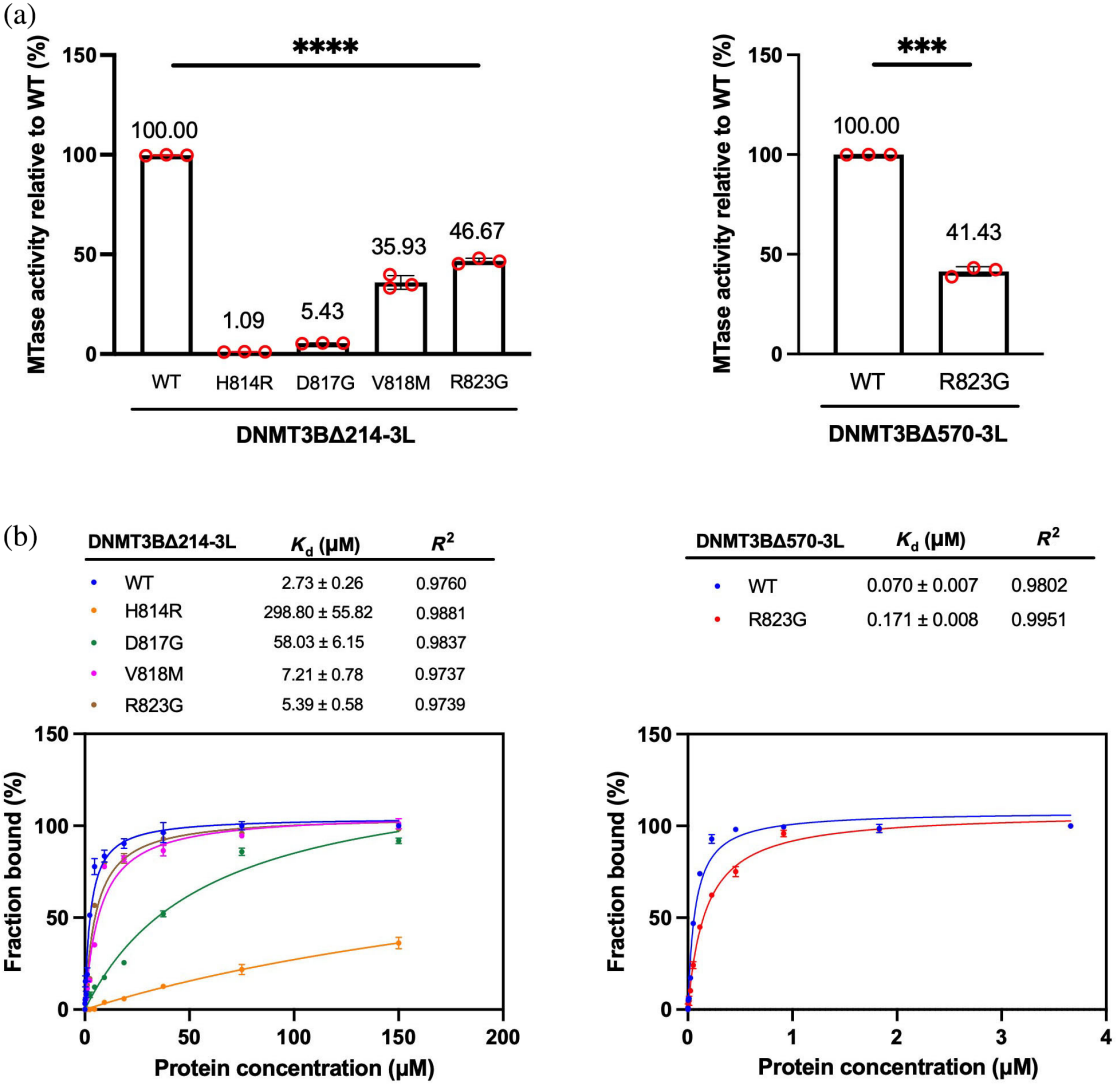
Immunodeficiency, centromere instability, facial anomalies (ICF) syndrome‐linked mutations in DNA methyltransferase 3B (DNMT3B) dimeric interface impair the methylation activities and DNA‐binding affinities of DNMT3B‐3L complexes. (a) The methyltransferase activities of wild‐type (WT) and mutant DNMT3BΔ214‐3L complexes (H814R, D817G, V181M, and R823G) (left panel), as well as WT and mutant DNMT3BΔ570‐3L complexes (R823G) (right panel), were measured by MTase‐Glo assays. Data are presented as mean ± SD from three independent experiments. Statistical significance (*p*‐values) was determined by one‐way analysis of variance (left panel) or two‐tailed Student's *t*‐test (right panel) with **** indicating *p* < 0.0001 and *** indicating *p* < 0.001. (b) The DNA‐binding activities of WT and mutant DNMT3BΔ214‐3L complexes (H814R, D817G, V181M, and R823G), as well as WT and mutant DNMT3BΔ570‐3L (R823G) complexes, were assayed by fluorescence polarization spectroscopy. Data are plotted as mean ± SD from three independent experiments. Data were fitted using GraphPad Prism version 9.

To investigate if these mutations in the RD interface affect DNMT3B's enzymatic activity, we determined the MTase activity of WT and mutated DNMT3B‐3L complexes at the same concentration of 0.2 μM by means of MTase‐Glo assays using 36‐basepair (bp) double‐stranded DNA (dsDNA) containing multiple CpG sites as substrate. We observed that the mutations significantly reduced the MTase activity of DNMT3BΔ214‐3L, with the H814R, D817G, V818M, and R823G mutants exhibiting 1.09%, 5.43%, 35.93%, and 46.67% of the MTase activity of WT complex (set to 100%), respectively (Figure 4a). Similarly, the DNMT3BΔ570‐3L complex hosting the R823G mutation exhibited 41.43% of the MTase activity of the respective WT complex (Figure 4a). These reductions in MTase activity observed in the DNMT3BΔ214‐3L mutant complexes correlated with the percentages of residual hetero‐tetrameric conformations observed in our AUC assays, with the H814R mutant having the lowest enzymatic activity (1.09%) also displaying the smallest tetrameric population (1.5%), whereas the V818M and R823G mutants with the highest enzymatic activity (35.93% and 46.67%) retained the greatest proportion (28.3% and 94.7%) of intact tetramers. This outcome indicates that the residual MTase activity of each mutant is attributable to the residual levels of heterotetrameric enzyme.

To investigate if the loss of MTase activity is due to impaired DNA binding, we measured the DNA‐binding activities of WT and mutated DNMT3B‐3L complexes using fluorescence polarization assays on fluorophore‐labeled 49‐bp DNA as substrate. We determined that WT DNMT3BΔ214‐3L exhibits a dissociation constant (*K*_d_) of 2.73 ± 0.26 μM. The four mutations we assessed all reduced the DNA‐binding affinity of the respective mutant enzymes: H814R with a *K*_d_ of 298.80 ± 55.82 μM; D817G with a *K*_d_ of 58.03 ± 6.15 μM; V818M with a *K*_d_ of 7.21 ± 0.78 μM; and R823G with a *K*_d_ of 5.39 ± 0.58 μM (Figure 4b). The pattern of these DNA‐binding affinities is similar to that of MTase activities described above, with the H814R mutant having the lowest DNA‐binding affinity and least MTase activity (1.09%), and V818M and R823G having the highest DNA‐binding affinity and the greatest MTase activity (35.93% and 46.67%). These observations strongly suggest that the loss of MTase activity is correlated with the defects in DNA binding.

The R823G mutant within the DNMT3BΔ570‐3L complex also presented a higher *K*_d_ value of 0.171 ± 0.008 μM, i.e., ca. two‐fold lower DNA‐binding affinity than WT complex (*K*_d_ of 0.070 ± 0.007 μM). Thus, the ca. two‐fold reduced MTase activity elicited by the R823G mutation (41.43%, Figure 4a) is likely due to the decreased DNA‐binding affinity of the DNMT3BΔ570‐3L complex. Together, these results demonstrate that all four of the ICF‐related mutations in the RD interface we assessed significantly impair the DNA‐binding and enzymatic activities of DNMT3B‐3L complexes, albeit to varying extents.

### 2.3 Crystal structure of the DNMT3BΔ570(R570G)‐3L complex reveals impaired hydrogen bonding network within DNMT3B

**FIGURE 5:**
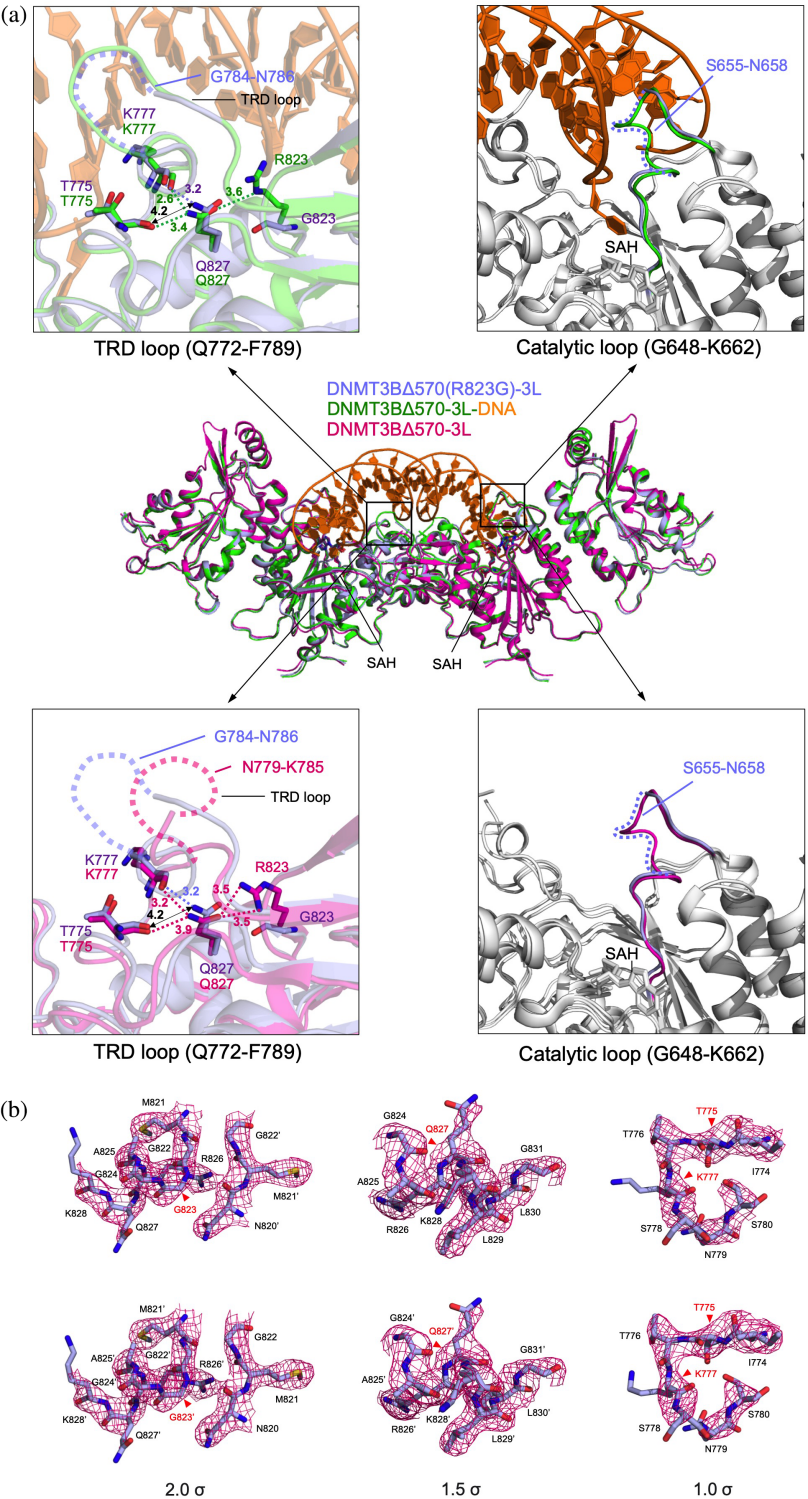
Crystal structure of the DNMT3BΔ570(R823G)‐3L tetrameric complex reveals impaired hydrogen bonding. (a) Superimposition of the mutant DNMT3BΔ570(R823G)‐3L structure (this study, PDB code: 8XEE, blue) with the wild‐type (WT) DNMT3BΔ570‐3L complex structure (PDB code: 6DKL, burgundy), and DNMT3BΔ570‐3L‐DNA complex structure (PDB code: 6KDA, green/brown) (Lin et al., 2020). The insets show enlarged views of the disordered TRD and catalytic loops in the mutant and WT complex. In the WT DNMT3B‐3L structure, R823 forms a hydrogen bond with Q827, which further interacts with T775 and K777 in the TRD loop (the hydrogen bonds are depicted as dashed lines). These hydrogen bonding interactions are lost or impaired in the R823G mutant. (b) The 2|*F*o| − |*F*c| omit maps around the mutated G823 (left panels, contoured at the 2.0 sigma level), Q827 (middle panels, contoured at 1.5 sigma level), and T775/K777 (right panels, contoured at 1.0 sigma level) of the two protomers are shown in red. DNMT3B, DNA methyltransferase 3B.

The R823G mutant complex primarily maintained a well‐folded tetrameric assembly (Figure 3), but its DNA‐binding and MTase activities were hampered (Figure 4). To better understand the defects elicited by the R823G mutation, we co‐crystallized the DNMT3BΔ570(R823G)‐3L complex with 24‐bp DNA and *S*‐adenosylhomocysteine (SAH). The X‐ray diffraction data to a resolution of 3.03 Å were collected using synchrotron radiations at the National Synchrotron Radiation Research Center, Taiwan. The crystal exhibited an identical space group and unit cell dimensions containing one hetero‐tetramer per asymmetric unit to the structures of the DNMT3BΔ570‐3L complex bound without and with DNA reported previously (PDB codes: 6DKL and 6KDA) (Lin et al., 2020). Two cofactor SAH molecules were bound in the DNMT3B homodimer as expected, at the identical cofactor binding site as observed in the previously reported crystal structures of DNMT3B‐3L complexes (Figure 5a) (Cho et al., 2023; Lin et al., 2020). However, despite multiple cycles of protein structure refinement, we could not observe an electron density for the DNA molecule, indicating that the DNA molecule was either not present or was disordered in the crystals. The arginine‐to‐glycine substitution at residue 823 was confirmed by the absence of the side‐chain density in the Fourier omit maps of both protomers (Figure 5b, left panels). The crystal structure of the DNMT3BΔ570(R823G)‐3L complex (PDB code: 8XEE) was refined to a final *R*‐factor and *R*‐free of 0.2212 and 0.2679 for 40,728 reflections (Figure 5a and Table 1).

**TABLE 1:** X‐ray diffraction and crystal structure refinement statistics for the DNMT3BΔ570(R823G)‐3L complex.

Data collection	
PDB entry	8XEE
Space group	P3_1_
Unit cell dimensions	
*a*, *b*, *c* (Å)	194.12, 194.12, 49.76
Wavelength (Å)	1.00
Resolution range (Å)	26.92–3.03 (3.13–3.03)
Unique reflections	40,728
Total reflections	212,477
Completeness^a^ (%)	99.3 (94.9)
*R*_merge_ ^a^, ^b^ (%)	0.06 (0.49)
*I*/*σ* ^a^	19.49 (3.48)
Redundancy^a^	5.2 (4.7)
CC_1/2_ ^a^	0.972 (0.906)
Refinement	
*R*_work_ (%)/*R*_free_ (%)^c^	22.12/26.79
R.M.S.D.	
Bonds (Å)	0.002
Angles (^o^)	0.51
Mean *B*‐factor (Å^2^)	71.59
Ramachandran plot (%)	
Favored	94.39
Allowed	5.06
Outliers	0.55

^a^ Values in parentheses are for the highest resolution shell.

^b^ *R*_merge_ = Σ_h_Σ_i_|*I*_h_,_i_ − *I*_h_|/Σ_h_Σ_i_ *I*_h_,_i_, where *I*_h_ is the mean intensity of the *i* observations of symmetry‐related reflections of *h*.

^c^ *R*_work_/*R*_free_ = Σ|*F*_obs_ − *F*_calc_|/Σ*F*_obs_, where *F*_calc_ is the calculated protein structure factor from the atomic model (*R*_free_ was calculated with 5% of the reflections selected).

Superimposition of the crystal structures of the DNMT3BΔ570(R823G)‐3L complex (PDB code: 8XEE) and WT DNMT3BΔ570‐3L bound without and with DNA (PDB code: 6DKL and 6KDA) (Lin et al., 2020) revealed high similarity, with an average RMSD of 0.83 and 0.31 Å, respectively, across 879 aligned C*α* atoms, supporting that the R823A mutation does not induce overall structural changes (Figure 5a). In the DNA‐free DNMT3BΔ570‐3L complex, two guanidinium nitrogen atoms (NH1 and NH2) of Arg823 form hydrogen bonds with the oxygen atom (Oε1) of Gln827, which further forms a hydrogen bond with the backbone carbonyl group of Thr775 and Lys777 located in the TRD DNA‐binding loop. However, the mutation of Arg823 to glycine abolished the hydrogen bond between Arg823 and Gln827, altering the side‐chain orientation of Gln827 and weakening its hydrogen bonding with Thr775 (the hydrogen bond distance increased from 3.9 to 4.2 Å) (see Figure 5b, bottom panels).

In the crystal structure of the DNA‐bound DNMT3BΔ570‐3L complex (PDB code: 6KDA), the Arg823 is located at the protein‐DNA interface, positioned proximal to the phosphate group of the +4 flank site (Lin et al., 2020). The substitution of the Arg823 with glycine may abolish the protein‐DNA interaction. Upon comparison with the DNA‐bound DNMT3BΔ570‐3L complex, the hydrogen bond between Arg823 and Gln827 is lost in the R823G mutant structure, resulting in altering the side‐chain orientation of Gln827 and weakening its hydrogen bonding with Thr775 (the hydrogen bond distance increased from 3.4 to 4.2 Å) and Lys777 (the hydrogen bond distance increased from 2.6 to 3.2 Å) (see Figure 5b, top panels). Lys777 is involved in making van der Waals interactions with the flanking guanine downstream of the CpG site in the WT structure (Lin et al., 2020), implying that the R823G mutation may affect the flanking sequence preference of DNMT3B. Similar results of a weakened hydrogen‐bonding network were also observed for the other protomer of the DNMT3B dimer (data not shown), confirming that R823G mutation induces structural changes at the protein‐DNA interface.

Moreover, upon comparison with the crystal structure of the WT DNMT3B‐3L‐DNA complex (PDB entry: 6KDA), it is evident that while the TRD and catalytic loops exhibit partial disorder in the mutant structure, they are well‐ordered within the protein‐DNA complex (Figure 5a). Upon comparison to the crystal structure of the DNA‐free DNMT3B‐3L complex (PDB entry: 6KDL), which was crystallized in the identical P3_1_ space group with isomorphous unit cell dimensions as those of the R823G mutant (PDB entry: 8XEE), the WT and mutant structures also display different flexibility in their respective DNA‐binding loops. The TRD loop (residues 772–789) in the WT DNMT3B is partially disordered from residues 781–786, but becomes less disordered in the R823G mutant from 784 to 786. On the other hand, the catalytic loop (residues 648–662) responsible for flipping out the cytosine at the DNA minor groove is well ordered in the WT DNMT3B, but becomes disordered between 655 to 658 in the R823G mutant for unknown reason. These findings shed light on the influence of the R823G mutation on the dynamics of the critical DNA‐binding TRD and catalytic loops. In summary, mutation of Arg823 to glycine not only disturbs protein‐DNA interactions but also affects the TRD loop conformation and dynamics as the hydrogen‐bonding network involving Thr775, Lys777, Arg823, and Gln827 within DNMT3B is impaired. Consequently, these changes diminish both the DNA‐binding and MTase activities of the protein.

### 2.4 The R823G mutation changes the flanking sequence preference of DNMT3B

**FIGURE 6:**
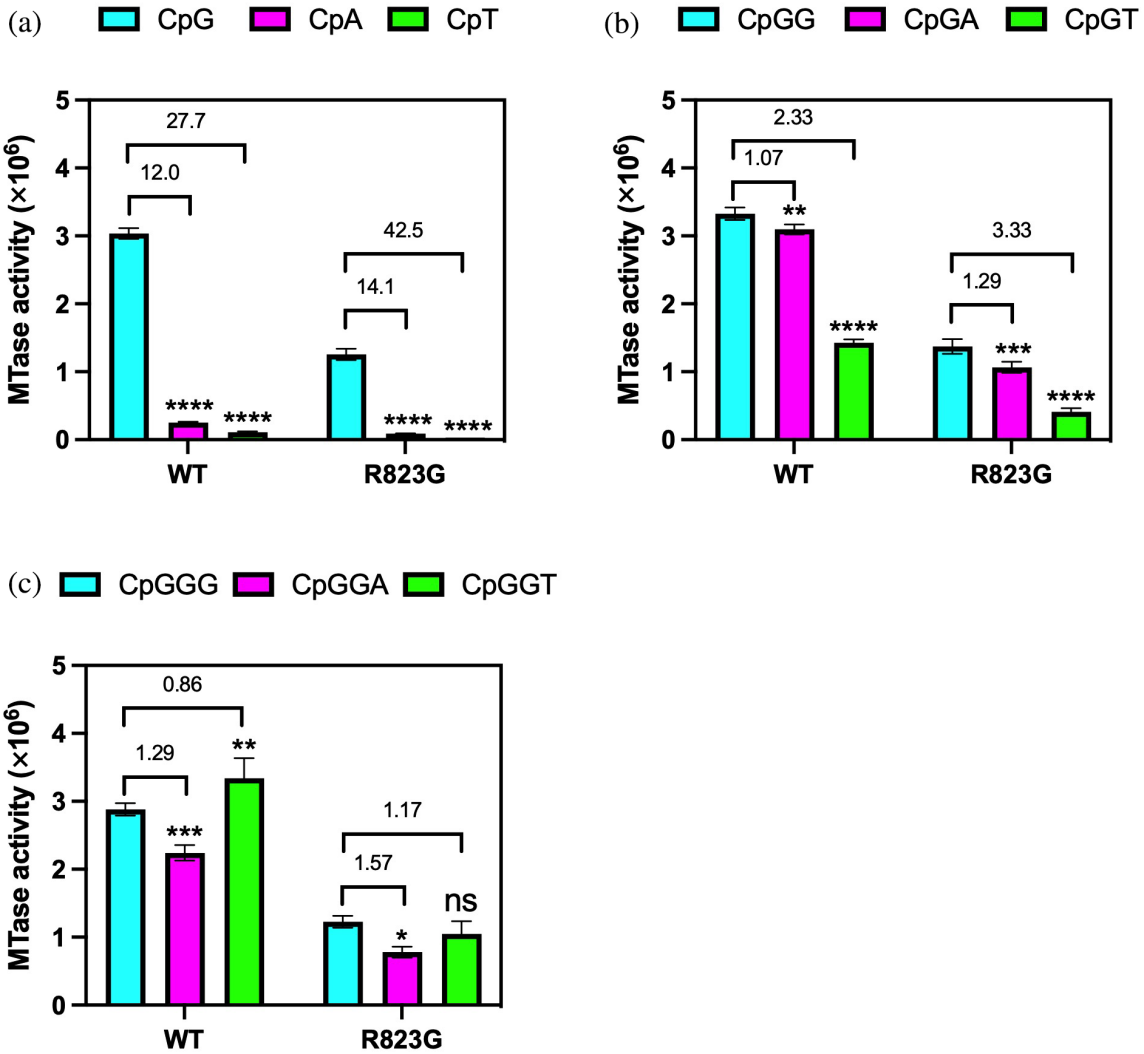
Flanking sequence preference is shifted upon R823G mutation of the DNMT3BΔ570‐3L complex. The methyltransferase activities of wild‐type DNMT3BΔ570‐3L complex (WT) and DNMT3BΔ570(R823G)‐3L mutant complex (R823G) in methylation of 24‐bp DNA substrate harboring (a) CpG, CpA, CpT, (b) CpGG, CpGA, CpGT, (c) CpGGG, CpGGA, or CpGGT sequences. The fold‐changes in methyltransferase activity of WT DNMT3BΔ570‐3L and the R823G mutant complex in methylating the DNA containing CpG/CpA, CpG/CpT, CpGG/CpGA, CpGG/CpGT, CpGGG/CpGGA and CpGGG/CpGGT are shown on the top of the bars (*n* = 3; error bars denote SD; *p < 0.1, **p < 0.01, *** <0.001, and **** <0.0001). Statistical significance (*p*‐values) was determined by two‐tailed Student's *t*‐test. DNMT3B, DNA methyltransferase 3B.

Based on the crystal structure of the DNMT3BΔ570(R823G)‐3L complex, it revealed that the R823G mutation might affect the flanking sequence preference of DNMT3B by shifting the orientation of Lys777 which is involved in interacting with the flanking guanine downstream of the CpG site (Lin et al., 2020). We therefore further assessed the sequence preference of the DNMT3BΔ570(R823G)‐3L complex in terms of methylating 24‐bp DNA substrate containing CpG, CpA, or CpT. The methylation activities were measured by MTase‐Glo assays and shown in Figure 6. We found that the WT enzyme methylated CpG with 12.0‐fold greater activity over CpA, and 27.7‐fold higher activity than determined for CpT, whereas the R823G mutant methylated CpG with 14.1‐fold greater activity than CpA, and 42.5‐fold higher activity relative to CpT, reflecting a shift in sequence preference at the +1 flanking position (Figure 6a).

We also assayed the flanking sequence preference using 24‐bp DNA containing CGN sequences (in which N represents G, A, or T at the +2 flanking position), or CGGN sequences (in which N represents G, A, or T at the +3 flanking position). Compared to the WT complex, the R823G mutant significantly increased the sequence preference at the +2 flanking position for CGG over CGT (3.33‐ vs. 2.33‐fold) and slightly increased the respective preference for CGG over CGA (1.29‐ vs. 1.07‐fold) (Figure 6b). For the +3 flanking position, the R823G mutant increased the sequence preference for CGGG over CGGA (1.57‐ vs. 1.29‐fold), and for CGGG over CGGT (1.17‐ vs. 0.86‐fold) (Figure 6c). These results support the notion that the R823G mutation of DNMT3B shifts the flanking sequence preference, rendering the enzyme more active at CGGG methylation sites with G located at the +1 to +3 flanking positions compared to WT DNMT3BΔ570‐3L complex.

## 3 DISCUSSION

DNMT3B is an essential enzyme that may function in concert with DNMT3L as a heterotetramer to establish DNA methylation patterns during embryonic development. DNMT3B forms a homodimer via the RD interface, and this homodimer further assembles with DNMT3L into a heterotetramer or with itself to form a homotetramer via the FF interfaces (Gao et al., 2022; Lin et al., 2020) (see Figure 1b). Stabilization of the RD interface in the DNMT3B homodimer therefore is critical to assembling the tetrameric DNMT3B‐3L and DNMT3B‐3B complexes. In this study, we found that mutations of H814R, D817G, and V818M in the RD interface severely impaired DNMT3B‐3L tetrameric assembly. Given that H814 of DNMT3B is involved in both aromatic stacking interactions (H814–H814′) and hydrogen bonding (H814–D817′) in the RD interface, it is perhaps not surprising that we found the H814R mutation to most seriously disrupt DNMT3B dimer assembly and MTase activity among the four mutants we tested, and that it leads to multiple syndromes, including not only the classical ICF phenotypes, such as the absence of IgA and IgG, respiratory infections, branching of chromosomes 1, 9, and 16, but also severe psychomotor retardation (Wijmenga et al., 2000). Notably, similar homologous mutations in DNMT3A, i.e., H873R in DNMT3A (equivalent to H814R in DNMT3B), and D876G in DNMT3A (equivalent to D817G in DNMT3B), also disrupt assembly of the DNMT3A‐3L tetramer and its DNA‐binding and MTase activities (Holz‐Schietinger et al., 2012). Collectively, these findings underscore the significance and imperative of preserving RD interface stability within both the DNMT3A and DNMT3B complexes.

Unlike the H814R, D817G, and V818M mutations, the R823G mutant variants of the DNMT3BΔ214‐3L and DNMT3BΔ570‐3L complexes largely maintain a tetrameric conformation (94.7% and 94.2%), yet their DNA‐binding affinity and MTase activity are reduced ca. two‐fold. The homologous R823 residue in DNMT3A (i.e., R882) is a hotspot mutational site in AML, with the R882H mutation representing the most frequent AML‐linked mutation among patients (Ley et al., 2010). Crystal structures of the MTase domain of the DNMT3A‐3L tetramer bearing the R882H mutation in complex with DNA of varying sequences have been reported previously, which reveal that the R882H mutation reduces DNA binding at the homodimeric interface and generates a more dynamic TRD loop (as verified by increased B factor values), as well as reduced MTase activities (Anteneh et al., 2020). These outcomes were different from our findings herein, demonstrating that the R823G mutation disrupts the hydrogen‐bonding network that involves Thr775, Lys777, Arg823, and Gln827 in the DNMT3B‐DNA interfaces. The disruption also changes the dynamics of the DNA‐binding TRD and catalytic loops in DNMT3B, leading to a consequential decline in DNA‐binding affinity and MTase activity.

Arg823 of DNMT3B was shown previously located closely to the DNA at the +4 flanking site in the crystal structure of the DNMT3B‐3L tetramer in complex with DNA (Gao et al., 2020). The R823G mutation weakens the hydrogen bonding between Q827 and K777, which is involved in sequence preference recognition of the flanking G sequence of the CGG site. Therefore, the R823 mutation may potentially change the flanking sequence preference of DNMT3B. A previous DNA sequence preference assay using deep enzymology revealed that the R823A mutant shares a similar overall methylation profile to WT DNMT3B (Dukatz et al., 2020). Nevertheless, a subset of sites featuring CGGG as the +1 to +3 flanking position exhibited superior methylation by the R823A mutant relative to WT DNMT3B (Dukatz et al., 2020). Our results further confirm the role of R823 in influencing the flanking sequence preferences of DNMT3B. Compared to WT enzyme, we found that the R823G mutant increases the preference to methylates CG, CGG, and CGGG sequences with G located at the +1 and +3 flanking positions. Hence, the R823G mutation compromises the enzyme via diverse mechanisms, with the effects extending beyond the evident reduction in DNA‐binding and MTase activities given that the R823G mutation also shifts the flanking sequence preference of DNMT3B, which could lead to off‐target CpG methylation.

In conclusion, our study furnishes compelling biophysical, biochemical, and structural evidence of the molecular mechanisms underlying how mutations in the RD interface of the DNMT3B homodimer may contribute to the onset of ICF syndrome. The H814R, D817G, and V818M mutations greatly perturb the formation of DNMT3B‐3L heterotetramers, consequently undermining their DNA‐binding and enzymatic activities. Furthermore, we have illuminated the structural foundation of defects attributed to another RD‐interfacial mutation, i.e., R823G. Our analysis of the crystal structure of the R823G mutant unveils a disrupted hydrogen‐bonding network at the protein‐DNA interface. These collective alterations ultimately promote a reduction in both DNA‐binding and methylation capabilities, as well as a shift in the flanking sequence preference of the enzyme. These ICF syndrome‐associated mutations in the RD interface trigger a multitude of anomalies, including perturbations in the intricate interplay between DNMT3B complex oligomerization and DNA interactions, resulting in the dysregulated DNA methylation observed in individuals affected by ICF syndrome.

## 4 METHODS

### 4.1 Protein expression and purification

The cDNA fragments encoding DNMT3BΔ214 (residues 215–853), DNMT3BΔ570 (residues 571–853), and DNMT3L (residues 179–379) were amplified by polymerase chain reaction. The DNA fragments encoding DNMT3BΔ214 were inserted into pSOL™ Expression Vectors (Lucigen), whereas the cDNAs encoding DNMT3BΔ570 and DNMT3L were cloned respectively into a modified pET28a(+) expression vector expressing an N‐terminal 6xHis‐SUMO tag and a tobacco etch virus (TEV) cleavage site, and an N‐terminal 6xHis‐tag and a TEV cleavage site to generate the pSol‐tev‐DNMT3BΔ214, pET28a(+)‐tev‐DNMT3BΔ570, and pET28a(+)‐tev‐DNMT3L plasmids. The H814R, D817, V818M, and R823G mutations were introduced into pSol‐tev‐DNMT3BΔ214, whereas the R823G mutation was introduced into pET28a(+)‐tev‐DNMT3BΔ570 plasmid, using a QuikChange™ site‐directed mutagenesis kit (Agilent).

All expression plasmids were transformed into the bacterial strain Rosetta2 (DE3) pLysS. Cells were grown at 37°C in LB Broth (Miller) medium containing 34 μg/mL chloramphenicol and 50 μg/mL streptomycin. Expression of DNMT3BΔ214, including WT enzyme, and the H814R, D817G, and V818M mutant variants, was induced by addition of 0.1% Rhamnose. In contrast, expression of DNMT3BΔ570 and the DNMT3BΔ570(R823G) mutant, as well as DNMT3L, was induced by 0.4 mM isopropyl‐thio‐β‐d‐galactoside until an OD_600_ of 0.6 had been attained. After induction, the cells were grown at 18°C overnight and harvested by centrifugation at 6000 rpm at 4°C for 30 min. The pellet was collected and re‐dissolved in lysis buffer containing 25 mM HEPES (pH 7.4), 500 mM NaCl, 5% glycerol, 0.5 mM tris(2‐carboxylethyl)phosphine (TCEP), and ethylenediaminetetraacetic acid‐free Protease Inhibitor Cocktail (Roche, Switzerland). The re‐dissolved soups of DNMT3BΔ214 or DNMT3BΔ570 were mixed with the re‐dissolved soup of DNMT3L in a 4‐to‐1 ratio to generate the DNMT3BΔ214‐3L (WT, H814R, D817G, V818M) and DNMT3BΔ570‐3L (WT and R823G) complexes.

The pellet mixture was lysed using a microfluidizer. After centrifugation at 17,000*g* for 45 min at 4°C, the supernatant was loaded through a HisTrap FF column (GE Healthcare). After equilibrium with Nickel wash buffer containing 50 mM sodium phosphate (pH 8.0), 500 mM NaCl, 5% glycerol, 5 mM β‐mercaptoethanol, and 40 mM imidazole, the His‐tagged protein was eluted by Nickel elution buffer (same constitution as Nickel wash buffer) and up to 500 mM imidazole. The 6xHis‐tag was removed by TEV protease during dialysis overnight at 4°C against a buffer containing 50 mM sodium phosphate (pH 8.0), 300 mM NaCl, 5% glycerol, and 10 mM β‐mercaptoethanol. The dialyzed protein sample was loaded into a HisTrap FF column (GE Healthcare), and the flow‐through fractions containing the cleaved, untagged protein of interest were collected. To further purify the protein sample, HiTrap Heparin HP (GE Healthcare) and gel filtration (HiLoad 16/60 Superdex 200, GE Healthcare) columns were used sequentially. The final purified samples of the DNMT3B‐3L complexes were solubilized in gel filtration buffer containing 20 mM Tris–HCl (pH 7.4), 200 mM NaCl, 5% glycerol, and 0.5 mM TCEP.

### 4.2 CD spectroscopy

Far‐UV CD spectra from 260 nm to 190 nm were recorded on an AVIV CD Spectrometer (Aviv Biomedical Inc.). All measurements were carried out in a 1‐mm quartz cuvette at 25°C. The protein samples in 20 mM phosphate buffer (pH 7.4) were set to 0.4 μM for DNMT3BΔ214‐3L, DNMT3BΔ214(H814R, D817G, V818M or R823G)‐3L, DNMT3BΔ570‐3L, and DNMT3B Δ570(R823G)‐3L complexes. The CD spectra were processed by smoothing and baseline subtraction using means of built‐in AVIV software. Ellipticity values (*θ*) were converted to mean residue ellipticity ([*θ*] in deg cm^2^ dmol^−1^) according to:

$$\left[\theta \right]=\theta \times M/10\times c\times l$$

where *M* is the mean molecular mass of the amino acids, *l* is the light path length in centimeters, and *c* is the protein concentration in moles per liter. All experiments were performed in triplicate. Secondary structure compositions were estimated and analyzed using BeStSel (Micsonai et al., 2018). Thermal denaturation was carried out by monitoring CD signals at 222 nm from 10 to 95°C. Tm values were calculated using the maximum of the first derivative of the CD signal.

### 4.3 Size‐exclusion chromatography coupled to multi‐angle light scattering

Molecular weights of DNMT3BΔ214 (WT, H814R, D817G, V818M, or R823G)‐3L were measured by means of SEC‐MALS. A Superdex Increase 200 10/300 column (GE Healthcare) was connected to a DAWN HELIOS II‐18 angle MALS (Wyatt Technology) detector with the wavelength set to 680 nm and equilibrated in 20 mM Tris–HCl (pH 8.0), 200 mM NaCl, and 1 mM TCEP with a flow rate of 0.2 mL/min using an ÄKTA‐UPC 900 FPLC system (GE Healthcare). Purified protein samples were centrifuged at 17,000*g* for 15 min at 4°C and filtered through a 0.22‐μm filter (Millipore). Protein samples (100 μL injection volumes) were injected into the Superdex column and then the UV fluorescence, MALS, and Refractive Index data were recorded and analyzed using ASTRA software (Wyatt Technology).

### 4.4 Analytical ultracentrifugation

Purified WT DNMT3BΔ214‐3L complex in a concentration of 2.5, 5.0, and 6.25 μM and the respective mutant complex (H814R, D817G, V818M, or R823G) (5 μM) were dialyzed in 20 mM Tris–HCl (pH 8.0), 300 mM NaCl, 1 mM TCEP and 5% glycerol overnight at 4°C. AUC analysis was carried out in the sedimentation velocity mode (SV‐AUC). A standard double‐sector centerpiece in an An‐60 Ti rotor was subjected to 40,000 rpm for 15 h at 20°C using a Beckman XL‐A analytical ultracentrifuge (Beckman Instruments, USA). The absorbance scans were monitored at 280 nm every 1.5 min interval. The solvent density (*ρ*) and viscosity (*η*) of the chemical components present in the buffer were calculated using SEDNTERP 3 (Philo, 2023) (Alliance Protein Laboratories). The AUC data were analyzed using the continuous distribution c(s) model in the program SEDFIT (Schuck, 2000).

### 4.5 MTase activity assay

The MTase activities of WT and mutant DNMT3BΔ214‐3L and DNMT3BΔ570‐3L complexes were measured using a MTase‐Glo™ Methyltransferase Assay Kit (Promega). The 36‐bp dsDNA substrate (2 μM with a sequence of 5′‐(GAC)_12_‐3′) was incubated with 0.2 μM protein in a reaction buffer containing 20 mM Tris–HCl (pH 8.0), 50 mM NaCl, 1 mM ethylenediaminetetraacetic acid, 1 mM DTT, 5% glycerol, 0.1 mg/mL bovine serum albumin, and 10 μM *S*‐adenosylmethionine in a volume of 4 μL at 37°C for 1 h. The reaction was stopped by adding 1 μL 0.5% trifluoroacetic acid for 5 min, followed by addition of 1 μL 6× MTase‐Glo™ reagent and incubated for 30 min. Next, 6 μL of MTase‐Glo™ detection reagent was added and incubated for another 30 min. The luminescence signals were recorded using an EnSpire Multimode Plate reader (PerkinElmer).

To analyze the flanking sequence preference of wild type and R823G mutant of DNMT3BΔ570‐3L complexes, 24‐bp complementary DNA harboring a CG, CA, CT, CGG, CGA, CGT, CGGG, CGGA, or CGGT sequence (2 μM):

5′‐AATT **CG** AAAAAATTTTTT **CG** AATT‐3′,

5′‐AATT **CA** AAAAAATTTTTT **TG** AATT‐3′,

5′‐AATT **CT** AAAAAATTTTTTA**G** AATT‐3′,

5′‐AATT **CGG** AAAAATTTTT **CCG** AATT‐3′,

5′‐AATT **CGA** AAAAATTTTT **TCG** AATT‐3′,

5′‐AATT **CGT** AAAAATTTTT **ACG** AATT‐3′,

5′‐AATT **CGGG** AAAATTTT **CCCG** AATT‐3′,

5′‐AATT **CGGA** AAAATTTT **TCCG** AATT‐3′,

5′‐AATT **CGGT** AAAATTTT **ACCG** AATT‐3′,

were incubated with WT or R823G mutant enzyme, respectively. MTase activity was measured using a MTase‐Glo™ Methyltransferase Assay Kit (Promega).

### 4.6 Determination of DNA‐binding affinity by fluorescence polarization assays

A 49‐bp 3′‐end FAM‐labeled dsDNA (10 nM) with a sequence of 5′‐AAAACGAAAAAAAAAAAAAAAAAAAAAAAAAAAAAAAAAAAAAAAAAAA‐3′ was incubated with increasing amounts of DNMT3BΔ214‐3L or DNMT3BΔ570‐3L complex for 30 min at 25°C in reaction buffer containing 20 mM Tris–HCl (pH 8.0), 50 mM NaCl, 5% glycerol, and 1 mM TCEP. Fluorescence polarization measurements were performed on a SpectraMax® Paradigm Multi‐Mode Detection Platform at 25°C and analyzed in SoftMax® Pro 7. The bound fractions were calculated as (mP − baseline mP)/(maximum mP − baseline mP), in which mP (milli‐polarization units) represents the fluorescence polarization value. The levels of protein–DNA complex formation were measured according to fluorescence polarization. Each reaction was performed in triplicate. The curves were fitted using GraphPad Prism 9.

### 4.7 Protein crystallization and structural determination

Purified human DNMT3BΔ570(R823G)‐3L complex (5.0 mg/mL), dsDNA (5′‐GAATTCGGAAAAATTTTTCCGAATT‐3′), and SAH were mixed in a molar ratio of 1:1.5:3. Protein crystals were grown by the hanging‐drop vapor diffusion method by mixing 1.6 μL of the DNMT3BΔ570(R823G)‐3L complex and 0.8 μL of reservoir solution containing 50 mM HEPES (pH 7.5), 10 mM MgCl_2_, 10% 2‐methyl‐2,4‐pentanediol, and 1.5 mM spermine. The crystals appeared after 1 week at 277 K and were soaked in a cryoprotectant composed of 25% glycerol and the reservoir solution before crystal mounting. The X‐ray diffraction data for the DNMT3BΔ570(R823G)‐3L complex were collected at TLS beamline 15A and TPS beamline 05A at the National Synchrotron Radiation Research Center, Hsinchu, Taiwan. The diffraction data were processed in HKL2000 (Otwinowski & Minor, 1997).

The crystal structure of the DNMT3BΔ570(R823G)‐3L complex was refined in PHENIX Phaser using the human WT DNMT3BΔ570‐3L complex structure (PDB code: 6KDA) as template. The initial model was rebuilt using the program Coot, and amino acid side chains and water molecules were fitted according to 2|*F*o| − |*F*c| and |*F*o| − |*F*c| electron density maps during repeated cycles of structural refinement in PHENIX (Liebschner et al., 2019). The data collection and structure refinement statistics are presented in Table 1.

## AUTHOR CONTRIBUTIONS

**Chao‐Cheng Cho:** Investigation; formal analysis; validation; writing – original draft; methodology; project administration. **Cheng‐Yin Fei:** Formal analysis; methodology; investigation. **Bo‐Chen Jiang:** Formal analysis; methodology; investigation. **Wei‐Zen Yang:** Methodology; investigation. **Hanna S. Yuan:** Conceptualization; formal analysis; writing – original draft; writing – review and editing; funding acquisition; project administration.

## FUNDING INFORMATION

This work was supported by Academia Sinica (post‐doctoral fellowship to C.C.C.) and the National Science and Technology Council of Taiwan ROC (MOST111‐2311‐B001‐011‐MY3).

## CONFLICT OF INTEREST STATEMENT

The authors declare no conflict of interest.
